# Streptococcal Extracellular Membrane Vesicles Are Rapidly Internalized by Immune Cells and Alter Their Cytokine Release

**DOI:** 10.3389/fimmu.2020.00080

**Published:** 2020-02-14

**Authors:** Mina Mehanny, Marcus Koch, Claus-Michael Lehr, Gregor Fuhrmann

**Affiliations:** ^1^Biogenic Nanotherapeutics Group, Helmholtz Institute for Pharmaceutical Research Saarland, Saarbrücken, Germany; ^2^Department of Pharmacy, Saarland University, Saarbrücken, Germany; ^3^Department of Pharmaceutics and Industrial Pharmacy, Faculty of Pharmacy, Ain Shams University, Cairo, Egypt; ^4^Leibniz-Institute for New Materials (INM) GmbH, Saarland University Saarbrücken, Saarbrücken, Germany; ^5^Drug Delivery Department, Helmholtz Institute for Pharmaceutical Research Saarland Saarbrücken, Saarbrücken, Germany

**Keywords:** extracellular membrane vesicles, *Streptococcus pneumoniae*, cytotoxicity, uptake, cytokine, vaccine

## Abstract

Extracellular vesicles are membranous structures shed by almost every living cell. Bacterial gram-negative outer membrane vesicles (OMVs) and gram-positive membrane vesicles (MVs) play important roles in adaptation to the surrounding environment, cellular components' exchange, transfer of antigens and virulence factors, and infection propagation. *Streptococcus pneumoniae* is considered one of the priority pathogens, with a global health impact due to the increase in infection burden and growing antibiotic resistance. We isolated MVs produced from the *S. pneumoniae* reference strain (R6) and purified them via size exclusion chromatography (SEC) to remove soluble protein impurities. We characterized the isolated MVs by nanoparticle tracking analysis (NTA) and measured their particle size distribution and concentration. Isolated MVs showed a mean particle size range of 130–160 nm and a particle yield of around 10^12^ particles per milliliter. Cryogenic transmission electron microscopy (cryo-TEM) images revealed a very heterogeneous nature of isolated MVs with a broad size range and various morphologies, arrangements, and contents. We incubated streptococcal MVs with several mammalian somatic cells, namely, human lung epithelial A549 and human keratinocytes HaCaT cell lines, and immune cells including differentiated macrophage-like dTHP-1 and murine dendritic DC2.4 cell lines. All cell lines displayed excellent viability profile and negligible cytotoxicity after 24-h incubation with MVs at concentrations reaching 10^6^ MVs per cell (somatic cells) and 10^5^ MVs per cell (immune cells). We evaluated the uptake of fluorescently labeled MVs into these four cell lines, using flow cytometry and confocal microscopy. Dendritic cells demonstrated prompt uptake after 30-min incubation, whereas other cell lines showed increasing uptake after 2-h incubation and almost complete colocalization/internalization of MVs after only 4-h incubation. We assessed the influence of streptococcal MVs on antigen-presenting cells, e.g., dendritic cells, using enzyme-linked immunosorbent assay (ELISA) and observed enhanced release of tumor necrosis factor (TNF)-α, a slight increase of interleukin (IL)-10 secretion, and no detectable effect on IL-12. Our study provides a better understanding of gram-positive streptococcal MVs and shows their potential to elicit a protective immune response. Therefore, they could offer an innovative avenue for safe and effective cell-free vaccination against pneumococcal infections.

## Introduction

*Streptococcus pneumoniae* (Pneumococcus) is a gram-positive bacterium, which normally colonizes the respiratory tract. It has invasive potential through mucosal membranes leading to severe diseases including otitis media, pneumonia, septicemia, and meningitis (1, 2). Young children and elderly populations, in addition to immunocompromised individuals, are the most prone to pneumococcal-related infections (3). These diseases account for high morbidities and mortalities worldwide, predominantly in developing countries (4, 5).

Pneumococcal infections are usually treated with several classes of antibiotics including penicillins, cephalosporins, macrolides, rifampin, and vancomycin (6). Pneumococcus, owing to its higher disease burden and increasing antibiotic resistance rates, poses a global health issue. Consequently, in 2017, the World Health Organization (WHO) announced *S. pneumoniae* in the list of priority pathogens, which require development of new antibiotic strategies (7). The introduction of a pneumococcal conjugate vaccine in 2000 decreased effectively the incidence of invasive streptococcal diseases (8). Nonetheless, it suffers from several shortcomings including incomplete protection, as it protects against only 23 capsular polysaccharide serotypes from 97 known serotypes (9). The rising prevalence of non-vaccine serotypes, due to genome remodeling by uptake and incorporation of exogenous DNA, is another principal limitation (2, 10). Therefore, the search for innovative safe and effective vaccination approaches against pneumococcal infections has never ceased.

Production of membrane vesicles (MVs) from gram-positive bacteria had been overlooked for decades, because it was assumed that their rigid thick cell wall would hinder shedding of membrane blebs (11). Gram-positive MVs (formerly denoted mesosomes) may date back to the 1970s but were considered as artifacts (12, 13). In addition to outer MVs (OMVs) from gram-negative bacteria, more light is shed recently on these MVs secreted from gram-positive bacteria including *Bacillus, Staphylococcus*, and *Streptococcus* species (14).

Bacterial vesicles could induce immune reactions in host cells (15, 16). They can interact with innate immune cells, e.g., macrophages and neutrophils, as well as adaptive immune cells and antigen-presenting cells (APCs), e.g., dendritic cells (DCs). Therefore, they may cause a protective immune response (17, 18). Activation of the immune system, upon introduction of pathogenic and/or its antigens or virulence factors, might elicit an immune response starting from innate immunity, which afterwards stimulates related adaptive immune cells, e.g., DCs (19). DCs could synthesize a broad pattern of cytokines, depending on applied stimuli to mature DCs, and demonstrate distinct driving potential for T helper cells (20, 21). The use of OMVs from gram-negative bacteria as vaccination approaches is well established and has been in clinical practice for several years. They proved to have acceptable safety and efficacy (22–25).

Overall, this motivated us to study pneumococcal MVs, assess their uptake and compatibility with mammalian cells, and evaluate their potential immunostimulatory effect and feasibility for a cell-free vaccine preparation. Extracellular MVs might constitute an excellent and innovative tool to impart protection against pneumococcal infections and a prospective vaccination avenue.

## Materials and Methods

### Microbial Culture and MV Isolation

*S. pneumoniae* reference strain R6 (ATCC® BAA255™, USA), whose genome is fully sequenced and annotated was selected (26). It lacks the capsule, as the capsular polysaccharide might diminish the yield of secreted MVs.

Bacteria were grown overnight in 1 L of Bacto™ brain heart infusion (BHI) medium (BD Biosciences) at 37°C/5% CO_2_ (Supplementary Figures 1, 2). The medium was then collected and centrifuged twice at 3,800 × g for 15 min at 4°C, in order to remove bacterial cells and fragmented debris. Afterwards, the collected supernatant was sterile filtered through Stericup Durapore PVDF 0.45-μm pore membrane filter (Merck Millipore, Germany). The sterility of the filtrate was checked via overnight incubation of the filtered supernatant, which has shown no bacterial growth upon inspection, as observed by the absence of any turbidity and no change in optical density at 600 nm.

The filtered supernatant was loaded into 70-ml ultracentrifuge tubes and centrifuged at 100,000 × g for 2 h at 4°C (rotor SW 45Ti, Optima L-90k, Beckman Coulter, Germany) to obtain a vesicle-rich pellet. Then the supernatant was removed carefully, and the pellet was resuspended in minimal volume (≈500 μl) of filtered phosphate buffer saline (PBS) (Gibco PBS tablets without calcium, magnesium, and phenol red).

### Purification

The resuspended pellet was purified by size-exclusion chromatography (SEC) column loaded with Sepharose CL-2B (GE Life Science, UK) to separate MVs from soluble protein impurities. Eluted fractions of 1 mL were collected in polypropylene tubes (Axygen, Corning, Germany) and then stored at 4°C for no longer than a week (27).

### Bicinchoninic Acid Assay (BCA)

To check the efficiency of SEC purification, protein concentration in collected SEC fractions was determined through a BCA kit (Sigma Aldrich), according to the manufacturer's recommendation. Analysis of samples was done in triplicates using a standard calibration curve of bovine serum albumin (BSA).

### Particle Size, Concentration, and Size Distribution

Particle size distribution and yield of MVs were determined using nanoparticle tracking analysis (NTA, LM-10, Malvern, UK). To achieve comparable results, samples were diluted up to 1:1,000 in filtered PBS to maintain vesicle concentration within the recommended range of 20–120 particles per frame. Briefly, a 100-μl MV sample was introduced into a green laser-illuminated chamber, and a high-sensitivity video with camera level 13–15 was captured; three videos of 30-s length were recorded and processed by NanoSight 3.1 software (28).

### Electron Microscopy

Cryogenic transmission electron microscopy (cryo-TEM) was performed on MVs pelleted after ultracentrifugation. Briefly, 5 μl of sample was dropped onto a holey carbon grid (type S147-4, Plano, Wetzlar, Germany) and plotted for 2 s before plunging into liquid ethane at *T* = −165°C using a Gatan (Pleasanton, CA, USA) CP3 cryo plunger. The sample was transferred under liquid nitrogen to a Gatan model 914 cryo-TEM sample holder and investigated at *T* = −173°C by low-dose TEM bright-field imaging using a JEOL (Tokyo, Japan) JEM-2100 LaB6 operating at 200-kV accelerating voltage.

Bacteria were prepared for scanning electron microscopy (SEM), by centrifugation at 1,000 × g for 5 min to pellet bacterial cells. The pellet was resuspended in 4% *p*-formaldehyde (PFA) to fix the cells for 1 h. Then bacterial cells were resuspended in 200 μl of hexamethyldisilazane (HMDS), and 2 μl suspension was mounted onto a silicon wafer and dried overnight. The samples were investigated with/without gold sputter coating, using an FEI Quanta 400 FEG (Thermo Scientific, USA) in high-vacuum conditions at 5-kV accelerating voltage.

### Preparation of Liposome and Polystyrene Bead Control Particles

Liposomes were prepared from dipalmitoylphosphatidylcholine (DPPC) and 1,2-dimyristoyl-*sn*-glycero-3-phosphocholine (DMPC) (molar ratio 3:2 and final concentration 5 mg/ml) using a thin-film hydration technique, followed by extrusion through a polycarbonate filter at 40°C. Liposomes were fluorescently labeled using the same protocol performed with MVs.

Commercially available polystyrene beads (Polybead® Carboxylate 0.1 μm, Polysciences, Inc., Germany) were used as inert nanoparticles.

### Cell Culture

Four cell lines were utilized in this work: A549 pulmonary carcinoma epithelial cell line (A549, DSMZ-ACC 107, Germany, RPMI 1640 supplemented with 10% fetal calf serum, FCS), HaCaT human keratinocyte cell line (HaCaT, CLS-300493, Germany, DMEM supplemented with 10% FCS), THP-1 human acute leukemia monocyte cell line (THP-1, DSMZ-ACC 16, Germany, RPMI 1640 supplemented with 10% FCS), and DC2.4 murine dendritic cell line (DC2.4, SCC142-Merck Millipore, Germany, RPMI 1640 supplemented with 10% FCS + 1x nonessential amino acids + 1x HEPES buffer + 0.0054x β-mercaptoethanol).

### Cytotoxicity Assessment of Pneumococcal MVs With Mammalian Somatic and Immune Cells

Two epithelial cell lines were used for the determination of viability and cytotoxicity, namely, A549 and HaCaT, and one immune cell line THP-1 (29).

A549 cells were seeded into a 96-well plate at a density of 20,000 cells per well and let to grow for 48 h, until 80–90% confluence. Similarly, HaCaT cells were seeded at a density of 40,000 cells per well for 48 h. THP-1 cells were seeded into 96-well plates at a density of 100,000 cells per well, after being differentiated with phorbol 12-myristate 13-acetate (PMA) at a concentration of 30 ng/ml and let to grow for 24 h (30).

Afterwards, old culture medium was aspirated and replaced with fresh phenol red-free RPMI 1640 medium, not supplemented with serum; then the cells were incubated for 24 h with dilutions of 10^3^-10^6^ purified MVs per cell. Controls were prepared as follows: cells treated with PBS (live cell control with 100% viability) and cells killed with 1% (w/v) Triton X (dead cell control with 0% viability).

One hundred microliters of supernatant was transferred to a new 96-well plate for further lactate dehydrogenase (LDH) assay (Cytotoxicity Detection Kit, Merck, Germany), which was utilized per manufacturer's instructions, and incubated with a reaction mixture for 10 min; then the absorbance was measured at 490 nm.

For viability assay, cells were washed with PBS, and 100 μl of fresh medium and 100 μl of 10% diluted PrestoBlue cell viability reagent (Thermo Fisher, Germany) were added. Then, cells were incubated for 30 min at 37°C, and fluorescence was measured (excitation wavelength 560 nm and emission wavelength 590 nm). Finally, viability was calculated compared to PBS- and 1% w/v Triton-X-treated controls (31).

### Cytotoxicity Evaluation of Pneumococcal MVs on DC2.4 Cells by Live–Dead Stain

DC2.4 cells were seeded in 24-well plates (3 × 10^5^ cells per well) and let to grow for 24 h 37°C/5% CO_2_. The medium was aspirated; then MVs were added onto cells at concentrations of 10^2^-10^5^ MV per cell and incubated for 24 h. PBS-treated cells were considered as live cells (negative control with 100% viability), whereas dead cells (positive control with 0% viability) were obtained through incubation with 4% PFA for 15 min at room temperature. Afterwards, cells were washed with Hank's Balanced Salt Solution (HBSS) buffer and detached using trypsin/EDTA and transferred to fluorescence-activated cell sorting (FACS) tubes and then pelleted by centrifugation at 300 × g at 4°C for 5 min. The cells were resuspended in HBSS buffer, were added with a 1-μl/tube reconstituted live/dead staining kit 568/583 (PromoCell, Germany), and were incubated at 4°C for 30 min. Then, we pelleted cells as mentioned before, washed them with HBSS buffer, and centrifuged them again to remove any excess stain. Eventually, the pellet is reconstituted in HBSS buffer and further analyzed using flow cytometry (LSRFortessa, BD Bioscience, USA). The dye can permeate the compromised cell membrane of dead cells and accumulate and label intracellular proteins, thus becoming highly fluorescent, in contrast with scarce penetration through the intact membrane of live cells, only labeling surface proteins and subsequently having much lower fluorescence signal than dead cells.

A threshold of 10,000 live cells was set, to be analyzed from forward scatter (FSC) vs. side scatter (SSC) gating, where phycoerythrin (PE-A) channel negative cells were considered as live cells, using FlowJo 10.6.1 software (FlowJo LLC, USA).

### Assessment of Uptake/Colocalization of Streptococcal MVs Within Mammalian Cells

Pelleted MVs after UC were fluorescently labeled by incubation with 2 μl of DiI (Vybrant DiI Cell-Labeling solution, Thermo Fisher, Germany) for 30 min at 37°C (32). Non-incorporated dye was separated through SEC, and the fractions with maximum fluorescence were used for further investigation.

A549 cells were seeded on 24-well plates (0.75 × 10^5^ cells per well), while HaCaT cells (1.25 × 10^5^ cells per well), and both cell lines were incubated for 48 h, until 80–90% confluence, while differentiated THP-1 (dTHP-1) cells (4 × 10^5^ cells per well) and DC2.4 cells (3 × 10^5^ cells per well) were seeded in 24-well plates. Then, both cell lines were let to grow for 24 h. Further, 100 μl of labeled MVs was added onto cells and incubated for 0.5, 2, and 4 h at 37°C/5% CO_2_. In addition, uptake of MVs into A549 and DC2.4 cells was tested after 24-h incubation at 37°C with 5% CO_2_.

Then the cells were washed three times with PBS (for A549 and HaCaT) and HBSS buffer (for dTHP-1 and DC2.4), to remove any residual MVs. Cells were then detached using trypsin/EDTA (for A549, HaCaT, and DC2.4 cells) and Accutase for (dTHP-1 cells). Then cellular suspensions were collected in FACS tubes and diluted with suitable buffers, i.e., 2% FCS in PBS (for A549 and HaCaT cells) and 2% FCS in HBSS (for dTHP-1 and DC2.4), until measurement with flow cytometry (LSRFortessa, BD Bioscience, USA) (33). We repeated the same procedure for all cell lines incubated at 4°C for 4 h, to determine the temperature dependence of MV uptake and mechanisms controlling it. Liposomes were used as control for uptake study and fluorescently labeled using the same protocol performed with MVs.

As mentioned before, a threshold of 10,000 live cells was set, to be analyzed from FSC vs. SSC gating. DiI-positive cells (PE-A channel) after successful uptake of DiI-labeled MVs was determined, as compared with PBS-treated cells (negative control), using FlowJo 10.6.1 software (FlowJo LLC, USA).

### Confocal Imaging of Cells

A549 cells were seeded (0.45 × 10^5^ cells per well) on eight-well imaging chamber plates (SPL Life Sciences, Korea), and HaCaT cells were also seeded (0.75 × 10^5^ cells per well); both were incubated for 48 h. In contrast, dTHP-1 cells (3 × 10^5^ cells per well) and DC2.4 cells (2 × 10^5^ cells per well) were incubated and let to grow for 24 h. Then, cells were treated with 100 μl of DiI-labeled MVs and then incubated for 0.5, 2, and 4 h at 37°C/5% CO_2_. In addition, colocalization of MVs into A549 and DC2.4 cells was tested, after 24-h incubation at 37°C with 5% CO_2_. After the supernatant was discarded, cells were washed twice with PBS and incubated with fluorescein–wheat germ agglutinin (Vector Laboratories, USA) at a concentration of 10 μg/ml for 15 min at 37°C/5% CO_2_, to stain membrane glycoproteins.

Subsequently, cells were washed twice with PBS and then fixed using 3.7% PFA for 20 min at room temperature. 4′,6-Diamidino-2-phenylindole (DAPI) (Life Technologies, Germany) was applied at a concentration of 1 μg/ml to stain cell nuclei for 15 min at room temperature. Then a few drops of mounting medium (Thermo, Germany) were added, followed by coverslips.

Images were captured using a Leica TCS SP8 confocal laser scanning microscope (CLSM, Leica Microsystems, Germany). Fluorescein was visualized with a 488-nm laser, DAPI with a 405-nm laser, and DiI with a 561-nm laser. A 20× water immersion objective lens, with resolution of 1,024 × 1,024, was used; furthermore, captured images were processed with LAS X software (LAS X 1.8.013370, Leica Microsystems, Germany).

### Detection of Cytokine Production From DC2.4 Cells Treated With Pneumococcal MVs Using ELISA

Determination of cytokine production was done using murine enzyme-linked immunosorbent assay (ELISA) kit (PeproTECH, USA). Briefly, special ELISA 96-well plates were coated overnight at room temperature with capture antibody. Nonspecific binding was blocked with block buffer (1% w/v BSA in PBS) for 1 h. Then the plates were incubated with sample and standard dilutions for 2 h. Cytokines present in the sample/standard were detected via detection antibody, incubated for 2 h. Signal amplification was employed using avidin conjugated to horseradish peroxidase (avidin–HRP), incubated with plates for 30 min. Eventually, plates were incubated with ABTS substrate solution and monitored at 5-min intervals for color development. Absorbance was measured at 405 nm, with wavelength correction at 650 nm. Controls for the assay included PBS-treated cells (as negative control) and lipopolysaccharide (LPS)-treated cells at a concentration of 10 μg/ml (as positive control). Two additional controls were conducted, namely, liposomes and polystyrene beads, for comparison with MVs.

### Statistical Analysis of Data

All data are shown as mean ± standard deviation (SD), where *n* is the number of independent experiments. All measurements were performed at least as independent triplicates. One-way analysis of variance (ANOVA) was applied to differentiate between groups, followed by suitable *post-hoc* tests.

## Results

### SEC Provides Efficient Purification of MVs From Protein Impurities

**Figure 1:**
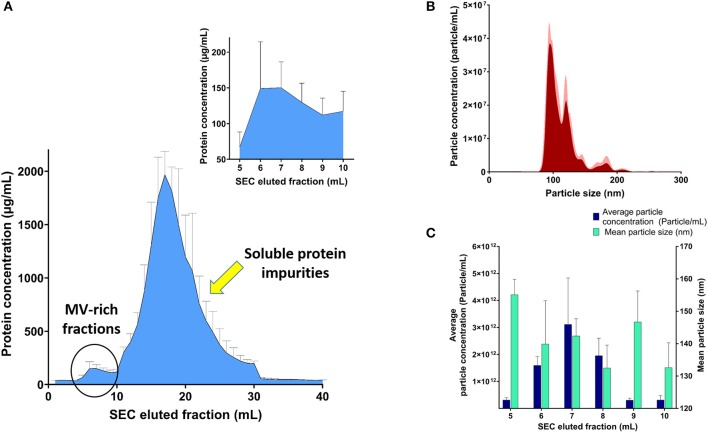
Analysis of streptococcal membrane vesicle (MV)-rich eluted fractions after size exclusion chromatography (SEC). **(A)** Bicinchoninic acid (BCA) assay of SEC eluted fractions with magnified part of MV-rich fractions in the upper-right corner. **(B)** Representative particle size–distribution curve of one of the vesicle-rich fractions (fraction 7), after suitable dilution for nanoparticle tracking analysis (NTA). **(C)** Graph representing average particle concentration (particle/mL) and mean particle size (nm) for the MV-rich SEC eluted 1-ml fractions, after NTA measurement. *n* = 3, mean ± SD.

SEC was used to purify the resuspended pellet after ultracentrifugation, without affecting their integrity, after collection of eluted 1-ml fractions after SEC purification and confirming efficient separation between MV-rich fractions and unwanted soluble protein impurities using BCA, as demonstrated in Figure 1A. Assessment of MV-containing fractions using NTA (Figures 1B,C) showed the highest concentration of particles in consecutive fractions 6, 7, and 8, in the range of 10^12^ particles per milliliter. We designated the MV concentrations to number of particles and not protein mass per fraction, because the conformational structure of surface proteins, ligands, and loaded cargo onto intact vesicles is more relevant to receptor binding and hence their biological effect. The isolated fractions exhibited average particle size ranging 130–160 nm, upon NTA analysis (Figure 1C).

### Pneumococcal MVs Display a Heterogeneous Nature

**Figure 2:**
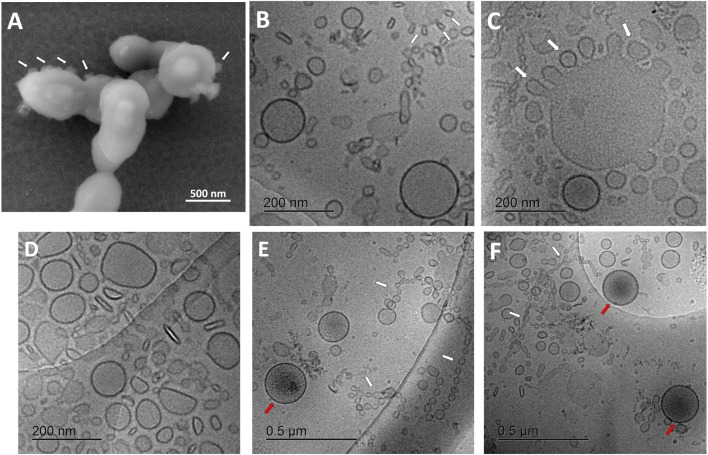
Characterization of streptococcal membrane vesicles (MVs) using electron microscopy. **(A)** Scanning electron micrograph (SEM) of *Streptococcus pneumoniae* reference strain R6 cells, during shedding of vesicles from their surface. **(B–F)** Cryogenic transmission electron micrographs (cryo-TEM) of isolated pneumococcal MVs, showing heterogeneous morphology, tiny vesicles budding from large ones (white arrows), chain-like structures (white arrows), and some vesicles with darker content (red arrows).

Visualization of streptococcus bacteria SEM (Figure 2A) revealed several protruding spherical particles, which are distinct from bacterial surface texture. They may show the process of vesicular release or budding through the bacterial cell wall.

For a better understanding of secreted pneumococcal MVs, cryo-TEM investigation was employed (Figures 2B–F). MVs exhibited a very heterogeneous nature, with respect to their morphology, particle size, arrangement, and content. Various structures and shapes were observed, such as spherical, elliptical, rod-shaped, and irregular forms (Figure 2D). In addition, many particles were aligned in chain-like structures (Figures 2E,F).

Interestingly, we also saw many smaller particles budding from larger structures (Figure 2C). Several particle sizes were recognized; the smallest were in the range of 20–30 nm in these chain-like assemblies, another population was almost 130–160 nm in mean diameter, and the largest reached around 300 nm in size. Some tiny vesicles budded from larger ones. Few MVs showed a darker color, suggesting a different content, rather than other vesicles (Figures 2E,F). SEC-eluted fractions showed some dilution effect (Supplementary Figure 3).

### Streptococcal MVs Demonstrate No Cytotoxicity With Somatic and Immune Cells

Assessment of cellular viability upon treatment with pneumococcal MVs and examination of whether they have any cytotoxic effects were performed on various mammalian cell lines.

**Figure 3:**
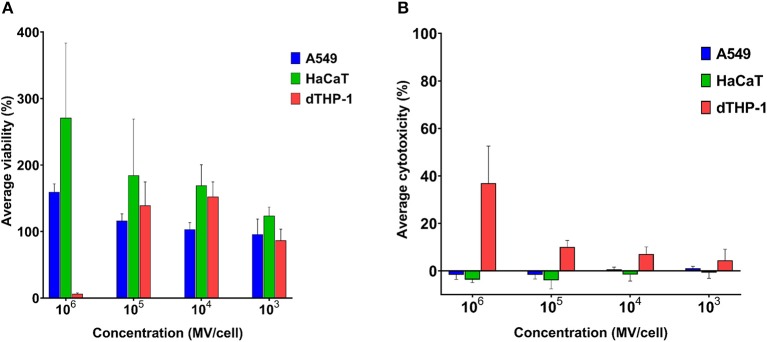
Streptococcal membrane vesicles (MVs) are noncytotoxic with somatic (i.e., A549 and HaCaT) and immune (i.e., dTHP-1) cell lines. **(A)** Calculated percentage viability of cells determined by PrestoBlue reagent and **(B)** calculated percentage cytotoxicity of cells determined by lactate dehydrogenase (LDH) reagent, in comparison with cells treated with PBS (live cell control) and 1% Triton X (dead cell control), after application of various dilutions of streptococcal MVs. *n* = 3, mean ± SD.

Mammalian somatic cells (A549 and HaCaT) exhibited very good tolerance to pneumococcal MVs. Even upon exposure to very high concentration of 10^6^ MVs per cell, cellular viability was not affected (Figure 3A).

Cellular viability was higher than positive control (100% viability) results. Interestingly, the higher the applied MV concentration was on cells, the higher was their calculated cellular viability. Pneumococcal MVs lacked any detectable cytotoxic effects on both cell lines (Figure 3B).

We examined MVs on dTHP-1 cells as a representative of innate immune cells. Concentrations (10^3^-10^5^ MVs per cell) caused no change in viability of cells, but calculated viability values were rather slightly higher than positive control. Besides these concentrations manifested inconsiderable toxic effects (below 10%). Only the highest concentration of MVs (10^6^ MVs per cell) compromised dTHP-1 cellular viability.

**Figure 4:**
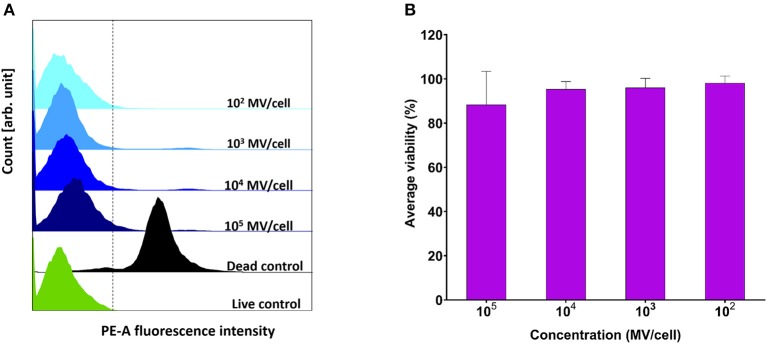
Viability assessment of dendritic cells (DC2.4) after exposure to pneumococcal membrane vesicles (MVs) by flow cytometry. **(A)** Overlay of recorded fluorescence intensity of phycoerythrin (PE-A) channel, after exposure of DC2.4 cells to various concentrations of MVs, in addition to live and dead cell controls. **(B)** Calculated percentage viability of DC2.4 cells with respect to cells treated with PBS (live cells control) and 4% PFA (dead cells control), after exposure to streptococcal MVs. The dashed line separates PE-A channel negative and positive cells. *n* = 3, mean ± SD.

Various concentrations (10^2^-10^5^ MVs per cell) were applied on DC2.4 cells. They showed comparable viability results to positive control (PBS treated) (Figure 4A), indicating their *in vitro* compatibility with adaptive immune cells and absence of any cytotoxic effects (Figure 4B).

### Streptococcal MVs Successfully Colocalize Within Mammalian Somatic and Immune Cells After a Relatively Short Incubation Period

**Figure 5:**
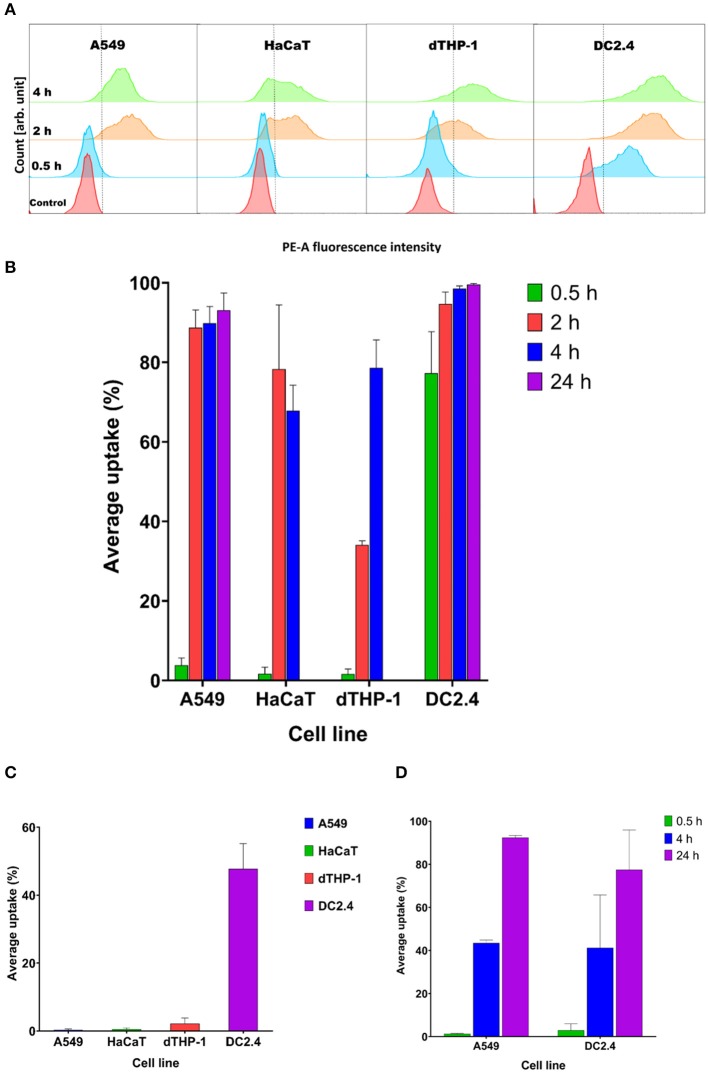
Streptococcal membrane vesicles (MVs) show successful uptake within mammalian cells shown by colocalization during flow cytometry measurement. **(A)** Phycoerythrin (PE-A) channel fluorescence intensity after incubation of cells with DiI-labeled MVs, with respect to control (PBS-treated) cells. The dashed line separates PE-A channel positive and negative cells. **(B)** Average percentage uptake of fluorescently labeled MVs inside four mammalian cell lines (A549, HaCaT, dTHP-1, and DC2.4) after different incubation periods (0.5, 2, and 4 h) and uptake of MVs into (A549 and DC2.4) cells after 24-h incubation. **(C)** Average percentage uptake of fluorescently labeled MVs into four mammalian cell lines (A549, HaCaT, dTHP-1, and DC2.4) after a 4-h incubation period at 4°C. **(D)** Average percentage uptake of fluorescently labeled liposomes into (A549 and DC2.4) cells after various incubation periods (0.5, 4, 24 h). *n* = 3, mean ± SD.

There was a prompt uptake of MVs by DC2.4 cells, with almost 80% of cells exhibiting uptake of fluorescently labeled MVs after only 30 min of incubation, in comparison with minimal uptake within other examined cell lines such as A549, HaCaT, and dTHP-1 (Figures 5A,B).

After 2 h of incubation, dTHP-1 cells showed a slow increase in uptake of MVs, reaching approximately 35%. While other cell lines demonstrated faster and higher uptake values (more than 80% for somatic A549 and HaCaT cells and 95% for immune DC2.4 cells).

Uptake readings of MVs for somatic cells level off after 4 h and show no remarkable difference between 2- and 4-h uptake values. dTHP-1 reached almost 80% uptake after 4 h, confirming their gradual uptake of MVs. Whereas as expected, DC2.4 cells exhibited almost complete uptake of all fluorescent MVs after 4 h of incubation period. After 24 h incubation, almost complete uptake of fluorescent MVs was detected, in both A549 and DC2.4 cells, without major changes from 4-h uptake observations.

Only DC2.4 cells exhibited uptake of pneumococcal MVs after 4-h incubation at 4°C, while no uptake was detected in other cell lines at this temperature (Figure 5C)

Liposomal controls exhibited negligible cellular uptake into somatic (A549) or immune (DC2.4) cells within 30 min, followed by a gradual increase reaching around 45% fluorescent cells after 4 h of incubation and almost complete uptake after 24 h (Figure 5D).

**Figure 6:**
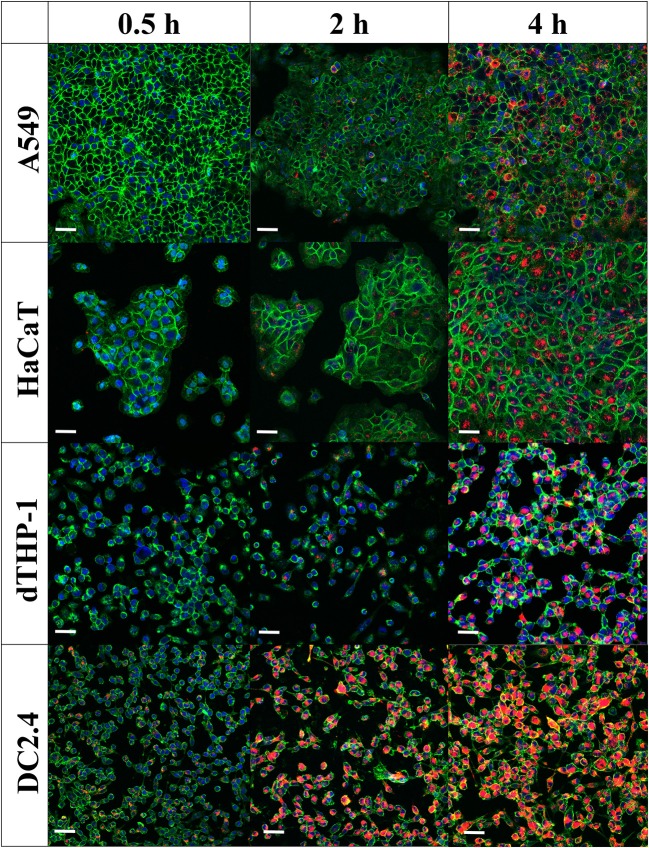
Internalization of pneumococcal membrane vesicles (MVs) into various somatic (A549 and HaCaT) and immune cell lines (dTHP-1 and DC2.4), as confirmed by confocal microscopy imaging, after various incubation periods (0.5, 2, and 4 h). Overlay images are shown, where nuclei are blue-stained [4′,6-diamidino-2-phenylindole (DAPI)], cellular membranes are green-stained [fluorescein–wheat germ agglutinin (WGA)], while MVs are orange-red-stained (DiI). Scale bar represents 50 μm.

Confocal laser scanning microscopy images (Figure 6) confirmed flow cytometry results for uptake of MVs into cells. The images showed minimal intracellular internalization of MVs after a 30-min incubation period, except for DC2.4 cells, which presented high and rapid uptake of MVs, as detected by the appearance of numerous orange-red dots of MVs fluorescently labeled with DiI within the green-stained cellular perimeter (Figure 6).

After 2 h of incubation, there is a gradual increase in MVs taken up by cells. In correlation with flow cytometry observations, dTHP-1 cells exhibited fewer red dots, in comparison with other cells. As anticipated, DC2.4 cells displayed more intracellular MVs, indicating the highest internalization of them into adaptive immune cells. All cell lines revealed abundant amounts of fluorescent MVs internalized especially after 4 h, confirming successful uptake of streptococcal MVs into mammalian cells (complete channels and overlay confocal images, in Supplementary Figures 4–6). No major change of MV colocalization into A549 and DC2.4 cells was detected, after 24-h incubation (Supplementary Figure 7).

### Streptococcal MVs Modulate Cytokine Production From Dendritic Cells

**Figure 7:**
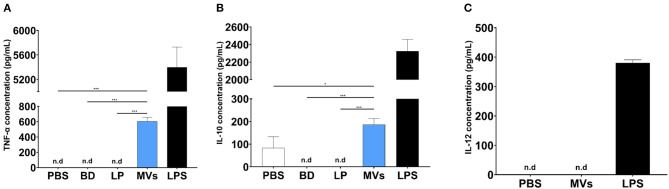
Pneumococcal membrane vesicles (MVs) possess immunomodulatory effect on antigen-presenting cells (APCs), e.g., dendritic cells (DC2.4). Phosphate buffer saline (PBS), polystyrene beads (BD), liposomes (LP), membrane vesicles (MVs), and lipopolysaccharide (LPS). Negative values are denoted not detected (n.d.) and considered zero value in statistical analysis. **(A)** Induction of TNF release, upon treatment of DC2.4 cells with pneumococcal MVs for a 24-h incubation period. **(B)** Slight increase of IL-10 release, upon treatment of DC2.4 cells with pneumococcal MVs for a 24-h incubation period. **(C)** Absence of any detected effect on IL-12 release, upon treatment of DC2.4 cell with pneumococcal MVs for 24-h incubation period. Differences between groups were determined using one-way analysis of variance (ANOVA) followed by (Sidak) *post hoc* test for multiple comparisons. *n* = 6, mean ± SD, where *P ≤ 0.05, ***P ≤ 0.0001.

Streptococcal MVs, after a 24-h incubation period with DC2.4 cells, could enhance the release of TNF-α (Figure 7A); a small increase in the levels of IL-10 (Figure 7B) and no detected effect on IL-12 release (Figure 7C) were observed. Increased secretion of TNF-α demonstrated an inflammatory effect and suggested a potential immunostimulatory effect upon application of MVs on DC2.4 cells.

## Discussion

In this work, we isolated and characterized streptococcal MVs to study their structure, interaction, and uptake into mammalian cells, as well as their potential for immunomodulatory effect. Our pneumococcal MVs showed average particle size, in the range of 130–160 nm as measured by NTA. This is comparable to what is reported by Surve et al. (34) for MVs from group B streptococci, who measured sizes of 150–300 nm using dynamic light scattering. However, Olaya-Abril et al. (35) described streptococcal vesicles, with particle size range of 20–75 nm, based on TEM image investigation.

A reason for this difference could be the purification technique employed, as Olya-Abril et al. utilized Optiprep density gradient fractionation. They reported that their vesicles might contain some cellular fragments or debris, due to lysis of apoptotic bacteria; hence, smaller-sized vesicles were observed. The difference in particle size obtained could arise from the difference in particle size measurement techniques, i.e., bulk NTA measurement vs. individual electron microscopy image analysis. In contrast, we applied SEC as our purification tool, which provides milder and better recovery of pure MVs, without compromising their purity, biological activity, or integrity (36, 37). It is worth mentioning that the lower size limit for NTA detection and measurement is 50 nm (38).

The differences in bulk size measurements in comparison to literature values motivated us to study the streptococcal vesicles by electron microscopy. Protrusions of tiny vesicles from bacterial cell wall as we observed them by SEM imaging may characterize the shedding process of pneumococcal MVs. This is in agreement with what was described earlier in literature regarding tiny blebs, appearing as rough protrusions distinct from the smooth surface texture of streptococci (34, 35). We furthermore detected a very broad particle size range of our isolated pneumococcal MVs, with four main populations of vesicles: (i) extremely small chain-like structures; (ii) tiny vesicles which bud from other vesicular forms with around 20- to 30-nm diameter both; (iii) medium-sized vesicle population of approximately 130–160 nm, also detected by NTA measurement; and (iv) a giant vesicular population from which the tiny ones bud, with an average diameter of 300 nm. Interestingly, the budding of tiny vesicles from giant counterparts was—to the best of our knowledge—not recorded earlier in literature in gram-positive MVs. These morphologies may suggest various biogenesis mechanisms involved in pneumococcal MV release (11). Characteristic chain-like structures (also known as nanotubes or nanowires) are protruding blebs from gram-positive bacterial cytoplasmic membrane, a phenomenon described also in gram-negative myxobacteria (39). They usually decorate cellular surface and may form membrane-bound networks to connect biofilm cells through periplasmic space. They may serve as bridges for exchange of cellular components (40, 41). In addition, a study on gram-negative *Vibrio vulnificus* showed that individual OMVs could detach from nanotubes, forming a unique “beads-on-a-string” pattern. OMVs were proposed to fuse to form nanotubes and *vice versa*, leading to nanotubes disintegrating into OMVs (42). This could justify the flexible nature of MV packing into chains and dismantling into separate vesicles according to the surrounding conditions as we observed and their importance for intercellular communication and exchange.

Two earlier studies (35, 43) performed detailed proteomic analysis of streptococcal MV components. They found that the majority of detected proteins were membrane-associated extracellular proteins (transmembrane and lipoproteins) or cytosolic proteins (since part of the cytoplasm is packed into MVs during biogenesis). Surface protein A (PspA) and maltodextrin-binding protein (malX) were identified by western blotting.

In a subsequent step, we were interested to study the impact of streptococcal MVs on various human and murine cell lines. MVs proved to be nontoxic toward mammalian somatic cells, namely, A549 and HaCaT cell lines. We used relatively high concentrations of MVs reaching 10^6^ MV per cell, without observing any detrimental effects on their viability. Streptococcal MVs conferred negligible cytotoxicity on both cell lines as examined by LDH assay. Choi et al. (43) reported similar results of pneumococcal MVs on A549 cells, suggesting they lack any major cytotoxic effect on mammalian somatic epithelial cells.

Surprisingly, cellular viability exceeded positive control (100%) the higher the applied MV concentration on cells was. MVs might be consumed by cells as a nutritional material rich in proteins and phospholipids, leading to a potential stimulatory effect on cell growth. Another possible reason we speculate is that MVs might activate cellular metabolic activity, resulting in stronger intracellular environment reducing resazurin, the PrestoBlue reagent, to fluorescent resorufin, leading to an increase in overall recorded fluorescence.

Subsequently, these findings were verified in cells involved in the potential immune response to MVs, namely, differentiated macrophage-like dTHP-1 cells as a representative of innate immune cells and DCs as the archetype of adaptive immune cells. Cellular viability for both cell lines (dTHP-1 and DC2.4) did not deteriorate upon treatment with concentrations (10^3^-10^5^ MV per cell). These concentrations did not display any negative effects on cells, proposing a potential noncytotoxic nature of pneumococcal MVs upon incubation with immune cells. dTHP-1 cells showed very good viability profile with all applied concentrations, except for the highest concentration (10^6^ MV per cell), which is an extremely high number of MVs. Nevertheless, we regarded pneumococcal MVs safe and suitable for further investigations.

We first studied the interaction and/or internalization of MVs within A549, HaCaT, dTHP-1, and DC2.4 cell lines. Pneumococcal MVs were successfully taken up and colocalized within different mammalian cells. DC2.4 cells could promptly internalize streptococcal MVs, reaching around 80% of positive cells after an incubation period of 30 min. This may suggest a specific receptor-mediated and/or endocytosis uptake mechanism for antigen-carrying MVs into APCs as has been reported in literature (44). There is negligible uptake of liposomes by DC2.4 cells after 30 min, suggesting a specific interaction between DC2.4 cells and streptococcal MVs.

Uptake of vesicles into mammalian cells might be a function of their particle size (45), as illustrated also by Turner et al. (46). They examined the mechanisms responsible for cellular entry of *Helicobacter pylori* OMVs. Smaller OMVs (20–100 nm) preferentially entered the cells through caveolin-mediated endocytosis, while larger OMVs (90–450 nm) transferred through micropinocytosis and endocytosis. Taking into account the mixed populations of MVs with larger and smaller sizes, the rapid uptake of streptococcal MVs into DCs after incubation for only 30 min is presumably due to mixed mechanisms. In literature, extracellular vesicle uptake relies mainly on endocytosis, phagocytosis, micropinocytosis, lipid raft internalization, and cell surface membrane fusion (33, 47, 48). Almost complete uptake of pneumococcal MVs in 4 h for DC2.4 cell line supports our hypothesis that MVs tend to be preferably taken up by DC2.4, owing to their role as APCs, in order to be processed and presented for further adaptive immune pathways. Interestingly, only DC2.4 cells demonstrated uptake of pneumococcal MVs upon incubation at 4°C, which might be ascribed to passive processes including lipid raft uptake through seemingly dynamic membrane areas enriched with sterols and sphingolipids, and membrane fusion of vesicles with cell surface (47, 48). In addition, we speculate that quick uptake by DCs might be beneficial, since they are the cells we want to target, for antigen processing and potential selective adaptive immune response.

These mixed uptake processes could explain the rapid uptake of MVs into DC2.4 cells after only 30-min incubation at 37°C. Further investigations will allow us to study the uptake kinetics of various MV populations and mechanisms controlling them.

Codemo et al. (49) examined internalization of pneumococcal infections in A549 after 24-h incubation and reported a concentration-dependent increase in intracellular localization of vesicles. Twenty-four-hour uptake results of streptococcal MVs into (A549 and DC2.4) cells exhibited no major changes from 4-h uptake, suggesting that MVs accumulate inside cells. This might be beneficial for sufficient processing of streptococcal antigens within immune APCs, e.g., DCs.

To further validate flow cytometry experiments, we performed additional confocal microscopy visualization of cellular internalization of DiI-labeled MVs. We found that approximately all DC2.4 cells have taken up fluorescent MVs, assuming that APCs have the highest rate in a very short incubation time (30 min) and extent (4 h) and that almost all cells showed MV uptake, in comparison with other examined cell lines. This suggests the ability of the immune system especially APCs (e.g., DC2.4) to recognize microbial MVs, in order to process them and elicit a protective immune response.

In a final step, the ability of MVs to modulate the interleukin release pattern of DCs was assessed. We observed increased secretion of TNF-α upon exposure of DC2.4 cells to streptococcal MVs. TNF-α is an endogenous alarm signal, which coordinates gene expression and cellular activity and drives inflammatory responses in injury or infection (50). It has a prominent role in regulation of immune cells and creates an inflammatory signal to recruit other immune cells to evoke an immunostimulatory cascade (51). A previous study used several pneumococcal strains (virulent serotype 4, TIGR4, and its isogenic mutants). They reported increased TNF-α production from human monocyte-derived DCs, after exposure to pneumococcal MVs (49). Their observations support our findings that pneumococcal vesicles display an immunomodulatory effect.

We believe that enhanced TNF-α secretion is necessary for an adequate inflammatory response from immune cells, which elicits an immune response afterwards (52). We noticed a slight increase in production of IL-10 from DC2.4 cells treated with pneumococcal MVs. IL-10 possesses a broad anti-inflammatory activity, chiefly on macrophages and DCs. It inhibits antigen presentation, decreases major histocompatibility complex class II expression, hinders DC differentiation from precursor monocytes and DC maturation, and decreases secretion of pro-inflammatory cytokines from immune cells (53). This observation might support our hypothesis that streptococcal MVs could modulate antigen presentation and immune response. Our results are similar to what was described by Codemo et al. They reported an increased release of IL-10 after pneumococcal MV exposure (49).

We observed no change in secretion of IL-12 from MV-treated DC2.4 cells. This cytokine is secreted mainly by activated APCs during antigen presentation. It functions as a bridge between innate and adaptive immune systems, as it aids in differentiation of naïve T cells into memory cell and cytokine-producing T helpers. It regulates several cellular pathways necessary for proper functioning of immune system, as it provides protection against infections (54). This result might be due to difference in activation of DCs upon exposure to live bacteria and their vesicles. This observation is in line with previously reported results, as they reported inability of pneumococcal MVs to induce secretion of IL-12 from DCs (49).

Our study provides a better understanding of MVs secreted from gram-positive streptococci. We isolated and purified streptococcal MVs and characterized them physicochemically. We exhibited a heterogeneous nature of streptococcal MVs and the presence of distinct populations, with respect to morphology, constitution, particle size, and content. Our findings confirmed rapid internalization of pneumococcal MVs within adaptive immune cells and rather slower uptake into somatic cells after relatively short incubation periods. This uptake pattern is optimal for interaction with immune cells. MVs altered cytokine release pattern from dendritic cells, indicating a possible immune interaction/response. Thus, streptococcal MVs could be investigated to develop a new avenue of safe and effective, cell-free vaccines against pneumococcal infections. Follow-up animal study should give enough evidence to confirm our *in vitro* findings.

## Data Availability Statement

The datasets generated for this study are available on request to the corresponding author.

## Author Contributions

MM conducted all experiments for MV isolation, characterization, and testing, developed figures, and analyzed experimental observations. MK captured electron microscopy images. C-ML helped with the study design and co-supervised the project with GF. GF conceived the overall study and wrote the main manuscript together with MM. All authors reviewed and approved the manuscript.

### Conflict of Interest

The authors declare that the research was conducted in the absence of any commercial or financial relationships that could be construed as a potential conflict of interest.

## References

[1] Mitchell AM Mitchell TJ Streptococcus pneumoniae: virulence factors and variation. Clin Microbiol Infect. (2010) 16:411–8. 10.1111/j.1469-0691.2010.03183.x 20132250

[2] Weiser JN Ferreira DM Paton JC. *Streptococcus pneumoniae*: transmission, colonization and invasion. Nat Rev Microbiol. (2018) 16:355–67. 10.1038/s41579-018-0001-8 29599457 PMC5949087

[3] Kelly SJ Taylor KB Li S Jedrzejas MJ. Kinetic properties of *Streptococcus pneumoniae* hyaluronate lyase. Glycobiology. (2001) 11:297–304. 10.1093/glycob/11.4.297 11358878

[4] Kadioglu A Weiser JN Paton JC Andrew PW. The role of *Streptococcus pneumoniae* virulence factors in host respiratory colonization and disease. Nat Rev Microbiol. (2008) 6:288–301. 10.1038/nrmicro1871 18340341

[5] Blasi F Mantero M Santus P Tarsia P. Understanding the burden of pneumococcal disease in adults. Clin Microbiol Infect. (2012) 18(Suppl 5):7–14. 10.1111/j.1469-0691.2012.03937.x 22882668

[6] Prado CAN Perloff S Pneumococcal infections (*Streptococcus pneumoniae*). Medsacpe. (2019). Available online at: https://emedicine.medscape.com/article/225811-overview (accessed September 02, 2019).

[7] WHO Global Priority List of Antibiotic-Resistant Bacteria to Guide Research, Discovery, and Development of New Antibiotics. Geneva: World Health Organization (2017). Available online at: https://www.who.int/news-room/detail/27-02-2017-who-publishes-list-of-bacteria-for-which-new-antibiotics-are-urgently-needed (accessed September 02, 2019).

[8] von Gottberg A de Gouveia L Tempia S Quan V Meiring S von Mollendorf C. Effects of vaccination on invasive pneumococcal disease in South Africa. N Engl J Med. (2014) 371:1889–99. 10.1056/NEJMoa1401914 25386897

[9] Geno KA Gilbert GL Song JY Skovsted IC Klugman KP Jones C. Pneumococcal capsules and their types: past, present, and future. Clin Microbiol Rev. (2015) 28:871–99. 10.1128/CMR.00024-15 26085553 PMC4475641

[10] Klugman KP. The significance of serotype replacement for pneumococcal disease and antibiotic resistance. Adv Exp Med Biol. (2009) 634:121–8. 10.1007/978-0-387-79838-7_11 19280854

[11] Brown L Wolf JM Prados-Rosales R Casadevall A. Through the wall: extracellular vesicles in Gram-positive bacteria, mycobacteria and fungi. Nat Rev Microbiol. (2015) 13:620–30. 10.1038/nrmicro3480 26324094 PMC4860279

[12] Silva MT Sousa JC Polonia JJ Macedo MA Parente AM Bacterial mesosomes. Real structures or artifacts? Biochim Biophys Acta. (1976) 443:92–105. 10.1016/0005-2736(76)90493-4 821538

[13] Bishop DG Work E. An extracellular glycolipid produced by *Escherichia coli* grown under lysine-limiting conditions. Biochem J. (1965) 96:567–76. 10.1042/bj0960567 4953781 PMC1207076

[14] Kim JH Lee J Park J Gho YS. Gram-negative and Gram-positive bacterial extracellular vesicles. Semin Cell Dev Biol. (2015) 40:97–104. 10.1016/j.semcdb.2015.02.006 25704309

[15] Becker A Thakur BK Weiss JM Kim HS Peinado H Lyden D. Extracellular vesicles in cancer: cell-to-cell mediators of metastasis. Cancer Cell. (2016) 30:836–48. 10.1016/j.ccell.2016.10.009 27960084 PMC5157696

[16] Hutcheson JD Aikawa E. Extracellular vesicles in cardiovascular homeostasis and disease. Curr Opin Cardiol. (2018) 33:290–7. 10.1097/HCO.0000000000000510 29465447 PMC5895489

[17] Kaparakis-Liaskos M Ferrero RL. Immune modulation by bacterial outer membrane vesicles. Nat Rev Immunol. (2015) 15:375–87. 10.1038/nri3837 25976515

[18] MacDonald IA Kuehn MJ. Offense and defense: microbial membrane vesicles play both ways. Res Microbiol. (2012) 163:607–18. 10.1016/j.resmic.2012.10.020 23123555 PMC3518640

[19] Iwasaki A Medzhitov R. Control of adaptive immunity by the innate immune system. Nat Immunol. (2015) 16:343–53. 10.1038/ni.3123 25789684 PMC4507498

[20] Morelli AE Zahorchak AF Larregina AT Colvin BL Logar AJ Takayama T Cytokine production by mouse myeloid dendritic cells in relation to differentiation and terminal maturation induced by lipopolysaccharide or CD40 ligation. Blood. (2001) 98:1512–23. 10.1182/blood.V98.5.1512 11520802

[21] Deatherage BL Cookson BT. Membrane vesicle release in bacteria, eukaryotes, and archaea: a conserved yet underappreciated aspect of microbial life. Infect Immun. (2012) 80:1948–57. 10.1128/IAI.06014-11 22409932 PMC3370574

[22] Bjune G Hoiby EA Gronnesby JK Arnesen O Fredriksen JH Halstensen A. Effect of outer membrane vesicle vaccine against group B meningococcal disease in Norway. Lancet. (1991) 338:1093–6. 10.1016/0140-6736(91)91961-S 1682541

[23] Boslego J Garcia J Cruz C Zollinger W Brandt B Ruiz S. Efficacy, safety, and immunogenicity of a meningococcal group B (15:P1.3) outer membrane protein vaccine in Iquique, Chile. Chilean national committee for meningococcal disease. Vaccine. (1995) 13:821–9. 10.1016/0264-410X(94)00037-N 7483804

[24] Goes A Fuhrmann G. Biogenic and biomimetic carriers as versatile transporters to treat infections. ACS Infect Dis. (2018) 4:881–92. 10.1021/acsinfecdis.8b00030 29553240

[25] Fuhrmann G Neuer AL Herrmann IK. Extracellular vesicles - a promising avenue for the detection and treatment of infectious diseases? Eur J Pharm Biopharm. (2017) 118:56–61. 10.1016/j.ejpb.2017.04.005 28396279

[26] Hoskins J Alborn WE Jr Arnold J Blaszczak LC Burgett S DeHoff BS. Genome of the bacterium *Streptococcus pneumoniae* strain R6. J Bacteriol. (2001) 183:5709–17. 10.1128/JB.183.19.5709-5717.2001 11544234 PMC95463

[27] Richter M Fuhrmann K Fuhrmann G Evaluation of the storage stability of extracellular vesicles. J Vis Exp. (2019) 147:e59584 10.3791/59584 31180350

[28] Frank J Richter M de Rossi C Lehr CM Fuhrmann K Fuhrmann G. Extracellular vesicles protect glucuronidase model enzymes during freeze-drying. Sci Rep. (2018) 8:12377. 10.1038/s41598-018-30786-y 30120298 PMC6098026

[29] Berger E Breznan D Stals S Jasinghe VJ Goncalves D Girard D. Cytotoxicity assessment, inflammatory properties, and cellular uptake of Neutraplex lipid-based nanoparticles in THP-1 monocyte-derived macrophages. Nanobiomedicine. (2017) 4:1849543517746259. 10.1177/1849543517746259 29942393 PMC6009795

[30] Lund ME To J O'Brien BA Donnelly S. The choice of phorbol 12-myristate 13-acetate differentiation protocol influences the response of THP-1 macrophages to a pro-inflammatory stimulus. J Immunol Methods. (2016) 430:64–70. 10.1016/j.jim.2016.01.012 26826276

[31] Schulz E Goes A Garcia R Panter F Koch M Muller R. Biocompatible bacteria-derived vesicles show inherent antimicrobial activity. J Control Release. (2018) 290:46–55. 10.1016/j.jconrel.2018.09.030 30292423

[32] Ofir-Birin Y Abou Karam P Rudik A Giladi T Porat Z Regev-Rudzki N. Monitoring extracellular vesicle cargo active uptake by imaging flow cytometry. Front Immunol. (2018) 9:1011. 10.3389/fimmu.2018.01011 29881375 PMC5976745

[33] Costa Verdera H Gitz-Francois JJ Schiffelers RM Vader P. Cellular uptake of extracellular vesicles is mediated by clathrin-independent endocytosis and macropinocytosis. J Control Release. (2017) 266:100–8. 10.1016/j.jconrel.2017.09.019 28919558

[34] Surve MV Anil A Kamath KG Bhutda S Sthanam LK Pradhan A. Membrane vesicles of group B streptococcus disrupt feto-maternal barrier leading to preterm birth. PLoS Pathog. (2016) 12:e1005816. 10.1371/journal.ppat.1005816 27583406 PMC5008812

[35] Olaya-Abril A Prados-Rosales R McConnell MJ Martin-Pena R Gonzalez-Reyes JA Jimenez-Munguia I. Characterization of protective extracellular membrane-derived vesicles produced by *Streptococcus pneumoniae*. J Proteomics. (2014) 106:46–60. 10.1016/j.jprot.2014.04.023 24769240

[36] Hong CS Funk S Muller L Boyiadzis M Whiteside TL. Isolation of biologically active and morphologically intact exosomes from plasma of patients with cancer. J Extracell Vesicles. (2016) 5:29289. 10.3402/jev.v5.29289 27018366 PMC4808740

[37] Ramirez MI Amorim MG Gadelha C Milic I Welsh JA Freitas VM. Technical challenges of working with extracellular vesicles. Nanoscale. (2018) 10:881–906. 10.1039/C7NR08360B 29265147

[38] Dragovic RA Gardiner C Brooks AS Tannetta DS Ferguson DJ Hole P. Sizing and phenotyping of cellular vesicles using nanoparticle tracking analysis. Nanomedicine. (2011) 7:780–8. 10.1016/j.nano.2011.04.003 21601655 PMC3280380

[39] Remis JP Wei D Gorur A Zemla M Haraga J Allen S. Bacterial social networks: structure and composition of *Myxococcus xanthus* outer membrane vesicle chains. Environ Microbiol. (2014) 16:598–610. 10.1111/1462-2920.12187 23848955 PMC4234120

[40] Baidya AK Bhattacharya S Dubey GP Mamou G Ben-Yehuda S. Bacterial nanotubes: a conduit for intercellular molecular trade. Curr Opin Microbiol. (2018) 42:1–6. 10.1016/j.mib.2017.08.006 28961452

[41] Toyofuku M Nomura N Eberl L. Types and origins of bacterial membrane vesicles. Nat Rev Microbiol. (2019) 17:13–24. 10.1038/s41579-018-0112-2 30397270

[42] Hampton CM Guerrero-Ferreira RC Storms RE Taylor JV Yi H Gulig PA. The opportunistic pathogen vibrio vulnificus produces outer membrane vesicles in a spatially distinct manner related to capsular polysaccharide. Front Microbiol. (2017) 8:2177. 10.3389/fmicb.2017.02177 29163452 PMC5681939

[43] Choi CW Park EC Yun SH Lee SY Kim SI Kim GH. Potential usefulness of *Streptococcus pneumoniae* extracellular membrane vesicles as antibacterial vaccines. J Immunol Res. (2017) 2017:7931982. 10.1155/2017/7931982 28210633 PMC5292160

[44] Jia J Zhang Y Xin Y Jiang C Yan B Zhai S. Interactions between nanoparticles and dendritic cells: from the perspective of cancer immunotherapy. Front Oncol. (2018) 8:404. 10.3389/fonc.2018.00404 30319969 PMC6167641

[45] Caponnetto F Manini I Skrap M Palmai-Pallag T Di Loreto C Beltrami AP. Size-dependent cellular uptake of exosomes. Nanomedicine. (2017) 13:1011–20. 10.1016/j.nano.2016.12.009 27993726

[46] Turner L Bitto NJ Steer DL Lo C D'Costa K Ramm G. *Helicobacter pylori* outer membrane vesicle size determines their mechanisms of host cell entry and protein content. Front Immunol. (2018) 9:1466. 10.3389/fimmu.2018.01466 30013553 PMC6036113

[47] Mulcahy LA Pink RC Carter DR. Routes and mechanisms of extracellular vesicle uptake. J Extracell Vesicles. (2014) 3. 10.3402/jev.v3.24641 25143819 PMC4122821

[48] French KC Antonyak MA Cerione RA. Extracellular vesicle docking at the cellular port: extracellular vesicle binding and uptake. Semin Cell Dev Biol. (2017) 67:48–55. 10.1016/j.semcdb.2017.01.002 28104520 PMC5484727

[49] Codemo M Muschiol S Iovino F Nannapaneni P Plant L Wai SN. Immunomodulatory effects of pneumococcal extracellular vesicles on cellular and humoral host defenses. MBio. (2018) 9:e00559-18. 10.1128/mBio.00559-18 29636428 PMC5893880

[50] Apostolaki M Armaka M Victoratos P Kollias G. Cellular mechanisms of TNF function in models of inflammation and autoimmunity. Curr Dir Autoimmun. (2010) 11:1–26. 10.1159/000289195 20173385

[51] Blanco P Palucka AK Pascual V Banchereau J. Dendritic cells and cytokines in human inflammatory and autoimmune diseases. Cytokine Growth Factor Rev. (2008) 19:41–52. 10.1016/j.cytogfr.2007.10.004 18258476 PMC2413068

[52] Schulke S. Induction of interleukin-10 producing dendritic cells as a tool to suppress allergen-specific T helper 2 responses. Front Immunol. (2018) 9:455. 10.3389/fimmu.2018.00455 29616018 PMC5867300

[53] Mosser DM Zhang X. Interleukin-10: new perspectives on an old cytokine. Immunol Rev. (2008) 226:205–18. 10.1111/j.1600-065X.2008.00706.x 19161426 PMC2724982

[54] Sun L He C Nair L Yeung J Egwuagu CE. Interleukin 12 (IL-12) family cytokines: Role in immune pathogenesis and treatment of CNS autoimmune disease. Cytokine. (2015) 75:249–55. 10.1016/j.cyto.2015.01.030 25796985 PMC4553122

