# Supplementary material for: Streptococcal Extracellular Membrane Vesicles Are Rapidly Internalized by Immune Cells and Alter Their Cytokine Release
Source: Front Immunol. 2020 Feb 14;11:80. doi: 10.3389/fimmu.2020.00080 (PMC7034238; doi:10.3389/fimmu.2020.00080)
Supplement: Supplementary file 1 [file Data_Sheet_1.docx]

**Streptococcal extracellular membrane vesicles are rapidly internalized by immune cells and alter their cytokine release**

Mina Mehanny^1,3,5^, Marcus Koch^4^, Claus-Michael Lehr^2,3^, Gregor Fuhrmann^1,3^*

^1^ Helmholtz Institute for Pharmaceutical Research Saarland, Biogenic Nanotherapeutics Group, Campus E8.1 - 66123 Saarbrücken, Germany

^2^ Helmholtz Institute for Pharmaceutical Research Saarland, Drug Delivery Department, Campus E8.1 - 66123 Saarbrücken, Germany

^3^ Department of Pharmacy, Saarland University, Campus E8.1 - 66123 Saarbrücken, Germany

^4^ Leibniz-Institute for New Materials (INM) GmbH, Saarland University, Campus D2.2 - 66123 Saarbrücken, Germany

^5^ Department of Pharmaceutics and Industrial Pharmacy, Faculty of Pharmacy, Ain Shams University - 11566, Cairo, Egypt

*corresponding author: phone +49 68198806-1500, email gregor.fuhrmann@helmholtz-hzi.de


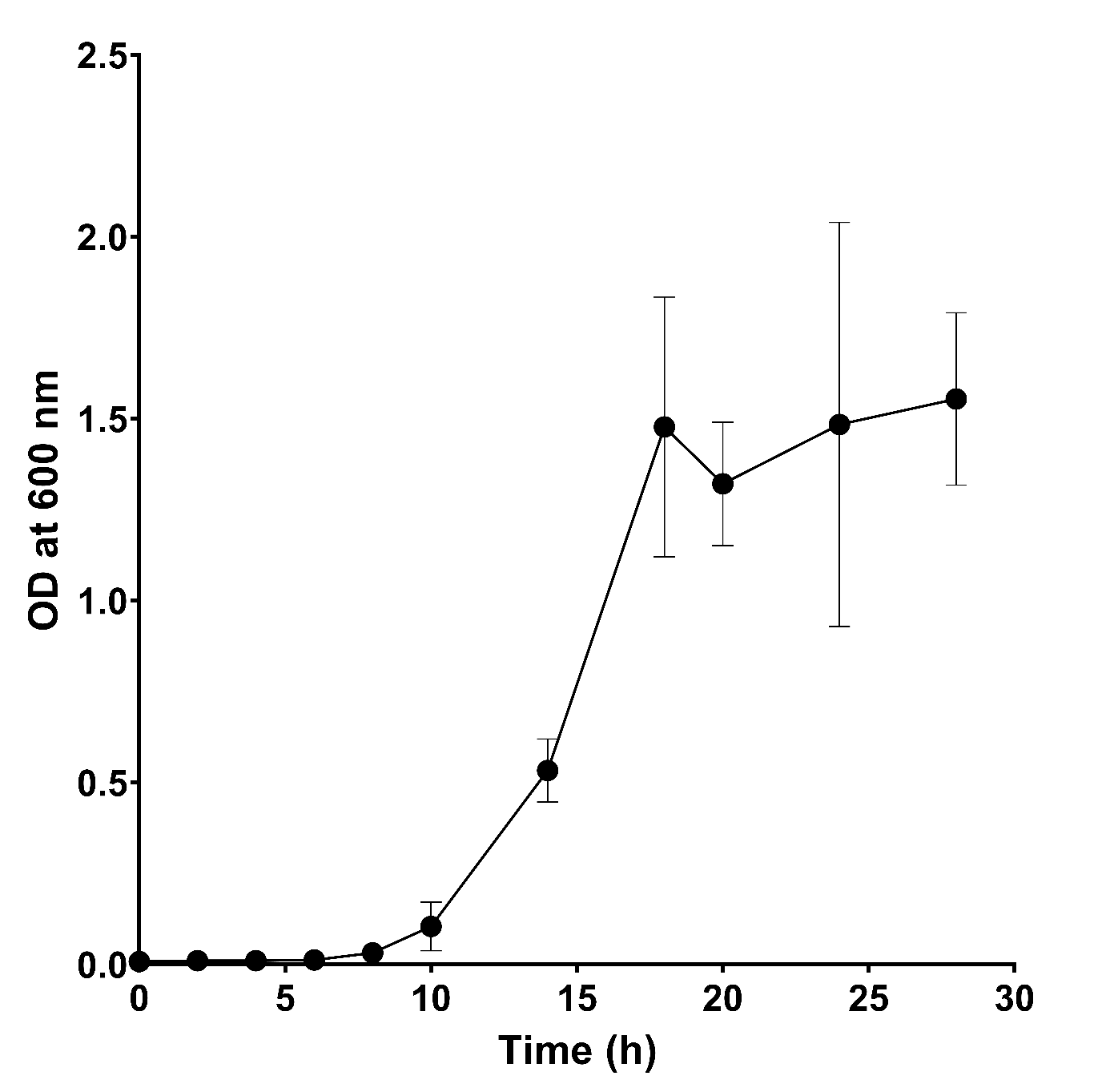


**Supplementary Figure 1.** Growth curve of *Streptococcus pneumoniae* reference strain R6, grown in brain heart infusion (BHI) medium


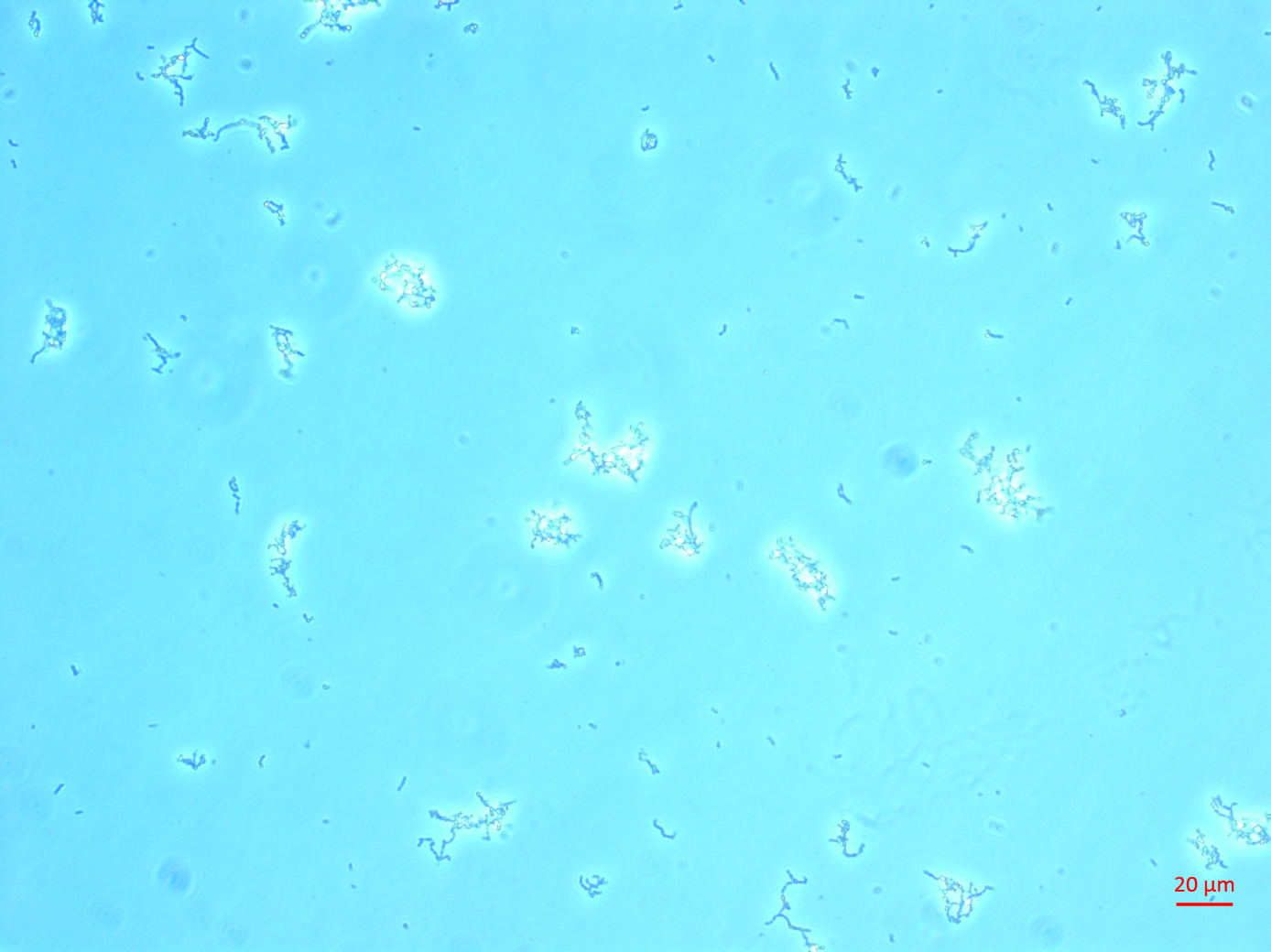


**Supplementary Figure 2.** Morphology of *Streptococcus pneumoniae* reference strain R6, grown in brain heart infusion (BHI) medium, under light microscope (32x magnification), in stationary phase (≈ 24 h)


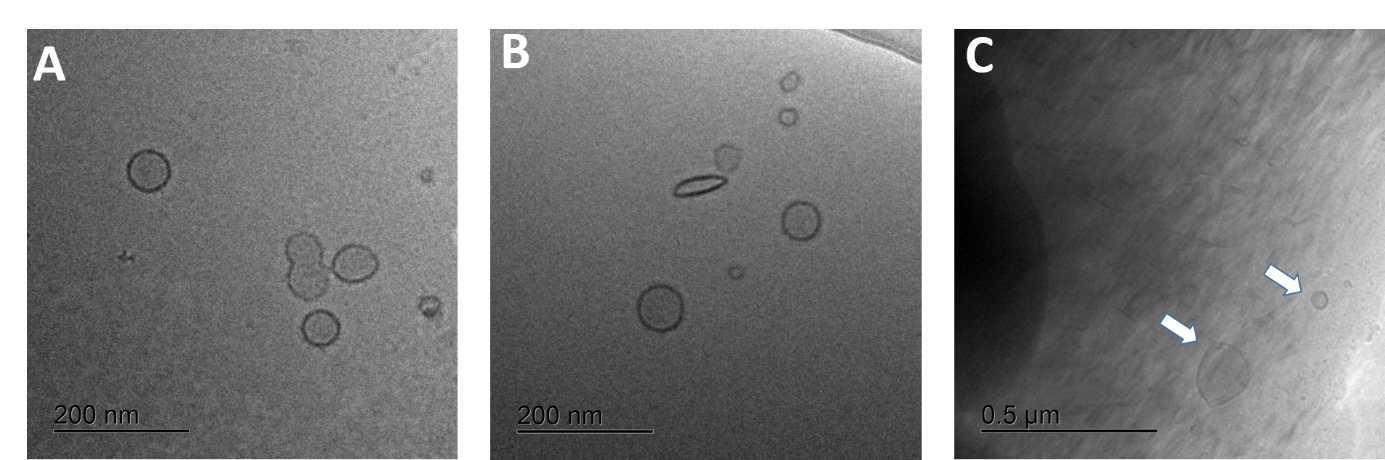


**Supplementary Figure 3.** Cryogenic transmission electron micrographs (cryoTEM) images of (A and B) Streptococcal membrane vesicles, eluted after purification with size exclusion chromatography (SEC) with some dilution effect. (C) Streptococcal bacterial cell with some vesicles shed near its surface.


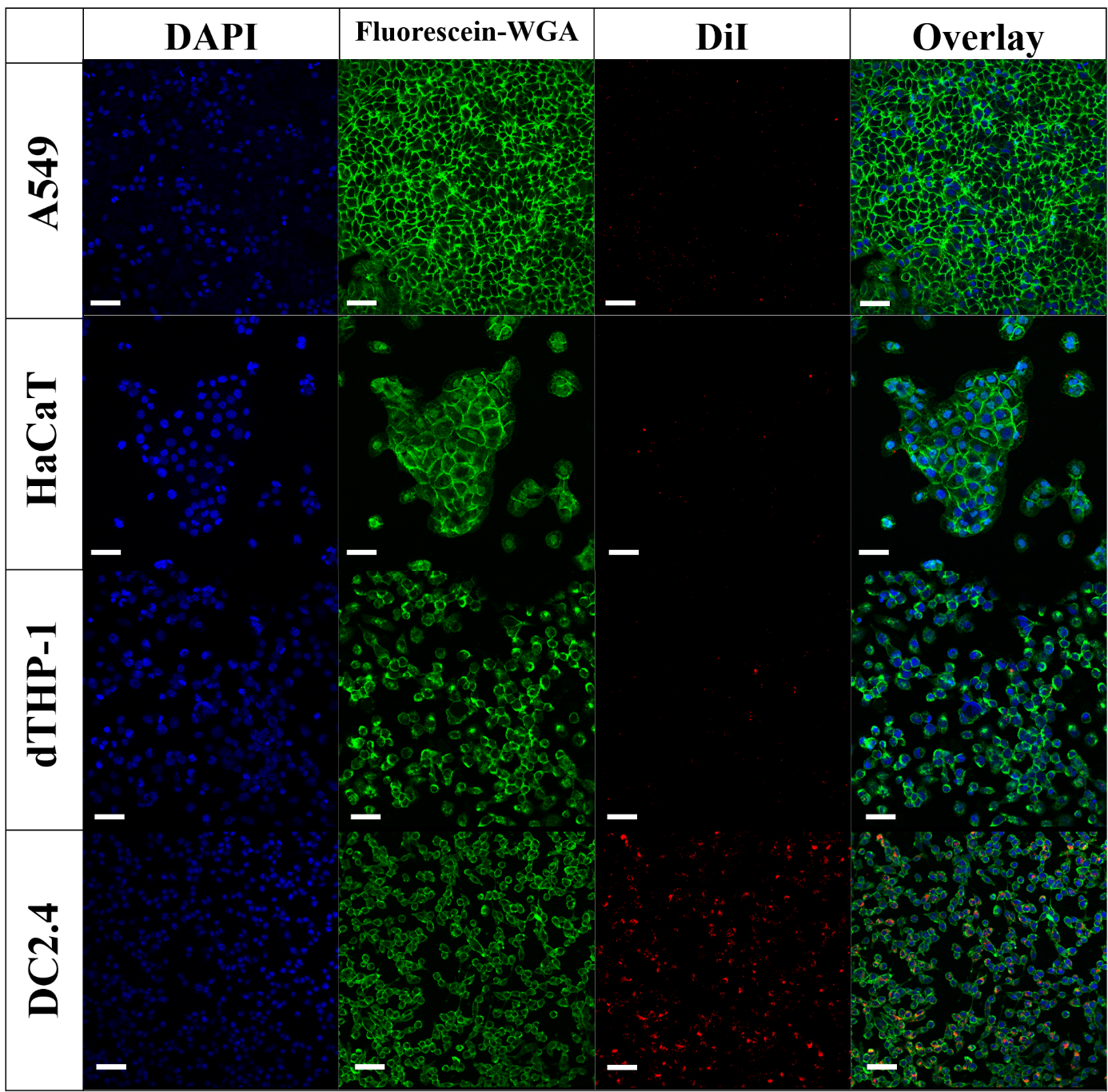


**Supplementary Figure 4.** Confocal laser scanning microscopy images of various somatic (A549 and HaCaT) and immune cell lines (dTHP-1 and DC2.4), showing internalization of DiI-fluorescently labelled streptococcal membrane vesicles (MVs) after 0.5 h incubation period. Scale bar represents 50 µm.


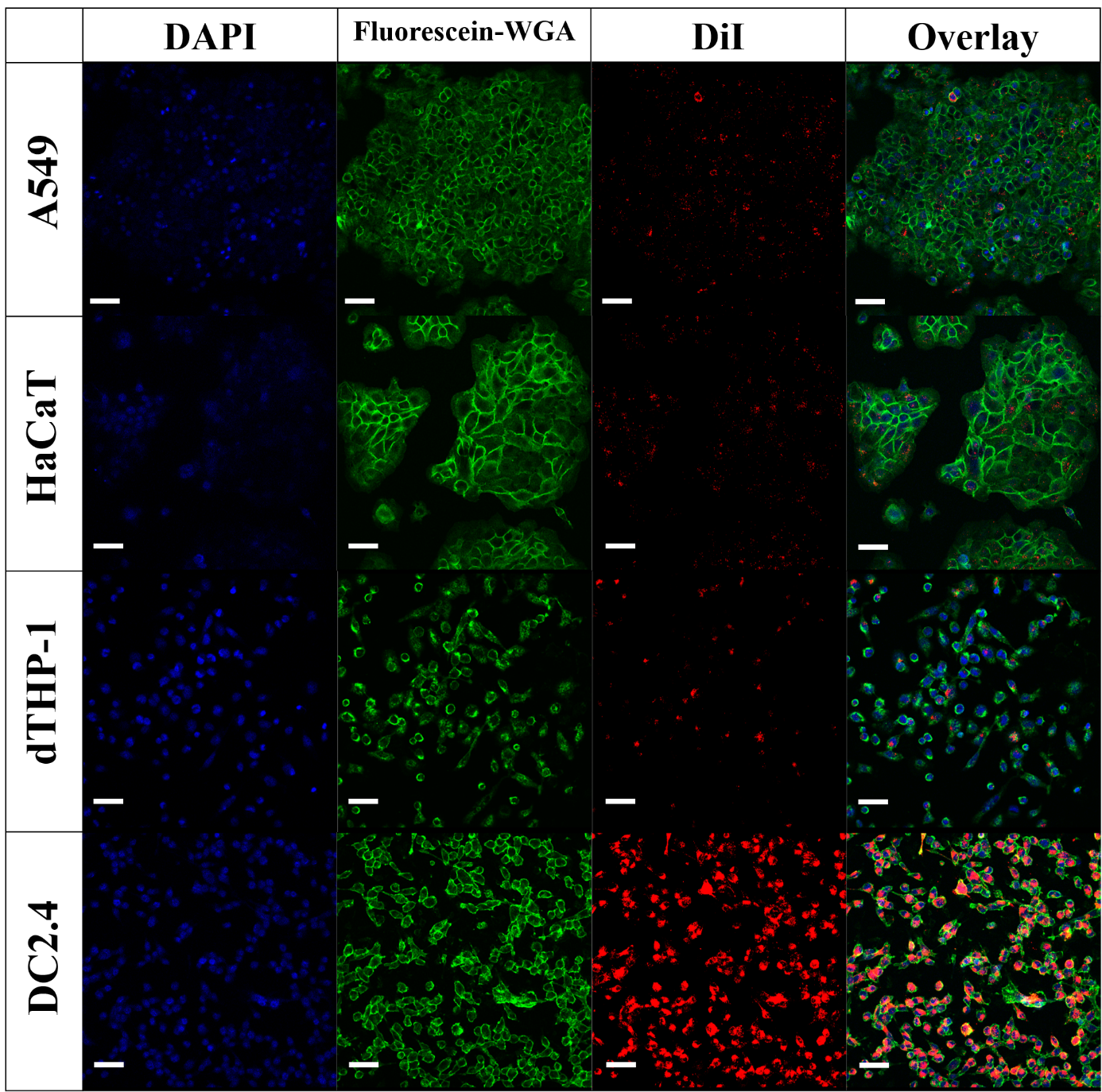


**Supplementary Figure 5.** Confocal laser scanning microscopy images of various somatic (A549 and HaCaT) and immune cell lines (dTHP-1 and DC2.4), showing internalization of DiI-fluorescently labelled streptococcal membrane vesicles (MVs) after 2 h incubation period. Scale bar represents 50 µm.


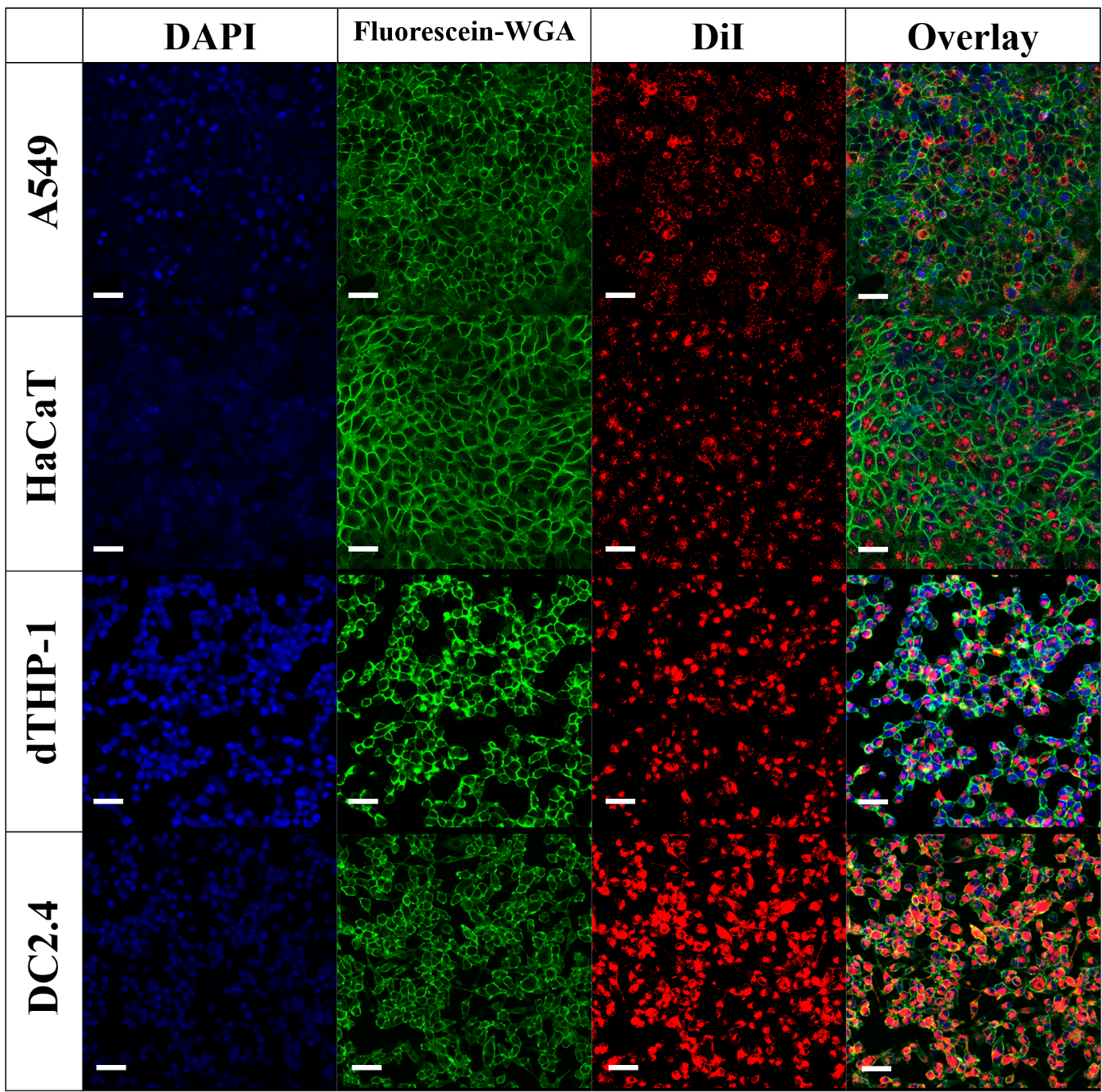


**Supplementary Figure 6.** Confocal laser scanning microscopy images of various somatic (A549 and HaCaT) and immune cell lines (dTHP-1 and DC2.4), showing internalization of DiI-fluorescently labelled streptococcal membrane vesicles (MVs) after 4 h incubation period. Scale bar represents 50 µm.


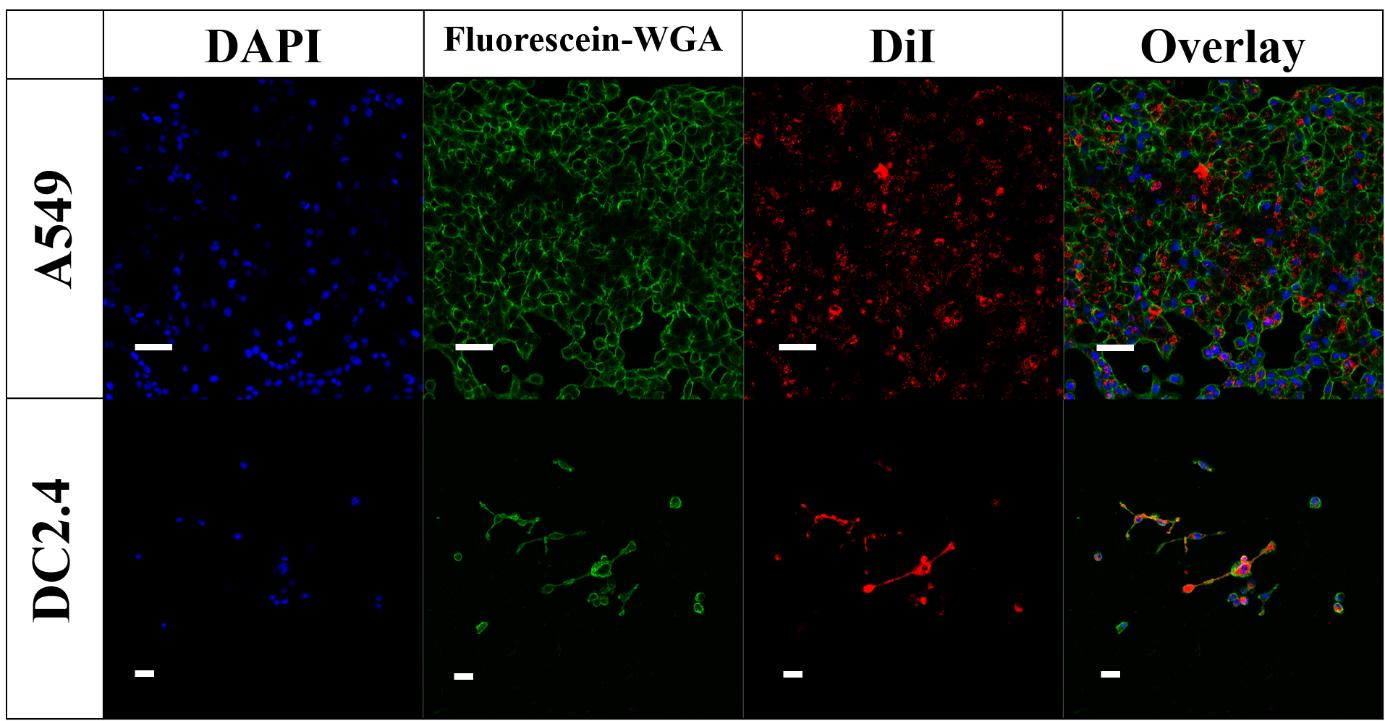


**Supplementary Figure 7.** Confocal laser scanning microscopy images of somatic (A549) and immune (DC2.4) cell lines, showing internalization of DiI-fluorescently labelled streptococcal membrane vesicles (MVs) after 24 h incubation period. Scale bar represents 50 µm.
